# Photoluminescent Histidine-Stabilized Gold Nanoclusters as Efficient Sensors for Fast and Easy Visual Detection of Fe Ions in Water Using Paper-Based Portable Platform

**DOI:** 10.3390/ijms232012410

**Published:** 2022-10-17

**Authors:** Alexandru-Milentie Hada, Markus Zetes, Monica Focsan, Simion Astilean, Ana-Maria Craciun

**Affiliations:** 1 Nanobiophotonics and Laser Microspectroscopy Center, Interdisciplinary Research Institute in Bio-Nano-Sciences, Babes-Bolyai University, 42 T. Laurian Str., 400271 Cluj-Napoca, Romania; 2 Faculty of Physics, Babes-Bolyai University, 1 M. Kogalniceanu Str., 400084 Cluj-Napoca, Romania

**Keywords:** gold nanoclusters, paper, photoluminescence, portable sensor, heavy metal ions, water

## Abstract

Herein is presented a novel and efficient portable paper-based sensing platform using paper-incorporated histidine stabilized gold nanoclusters (His-AuNCs), for the sensitive and selective detection of Fe ions from low-volume real water samples based on photoluminescence (PL) quenching. Highly photoluminescent colloidal His-AuNCs are obtained via a novel microwave-assisted method. The His-AuNCs-based sensor reveals a limit of detection (LOD) as low as 0.2 μM and a good selectivity towards Fe ions, in solution. Further, the fabricated portable sensing device based on paper impregnated with His-AuNCs proves to be suitable for the easy detection of hazardous Fe levels from real water samples, under UV light exposure, through evaluating the level of PL quenching on paper. Photographic images are thereafter captured with a smartphone camera and the average blue intensity ratio (I/I_0_) of the His-AuNCs-paper spots is plotted against [Fe^2+^] revealing a LOD of 3.2 μM. Moreover, selectivity and competitivity assays performed on paper-based sensor prove that the proposed platform presents high selectivity and accuracy for the detection of Fe ions from water samples. To validate the platform, sensing assays are performed on real water samples from local sources, spiked with 35 μM Fe ions (i.e., Fe^2+^). The obtained recoveries prove the high sensitivity and accuracy of the proposed His-AuNCs-paper-based sensor pointing towards its applicability as an easy-to-use, fast, quantitative and qualitative sensor suitable for on-site detection of toxic levels of Fe ions in low-volume real water samples.

## 1. Introduction

In the past few years, the pollution generated by heavy metal ions has been shown to represent an issue of great concern for the environment and human health, due to their high toxicity and non-biodegradable properties [1,2,3]. Particularly, even though iron (Fe) ions play an essential role in pathological and physiological processes, such as cell metabolism, oxygen transport and enzyme catalysis [4], they can be extremely harmful to the human body when accumulated in abnormal levels. The World Health Organization consider that concentrations of Fe ions as low as 35 μM in water can start to have unwanted effects for human health [5], inducing a variety of disorders including Parkinson’s and Alzheimer’s diseases, low blood pressure and many more [6]. There are plenty of methods for detecting unwanted concentrations of heavy metal ions, including Fe ions, for example chromatography [7], atomic absorption spectroscopy [8], electrochemical methods [9], surface enhanced Raman spectroscopy [10], inductively coupled plasma mass spectroscopy [11], etc. Yet, all of these sensing techniques share the same drawbacks related to their time- and money-consuming character, considering that the samples need to be moved to dedicated laboratories and institutions, where only trained personal using expensive equipment can perform the specific detection of the desired ions. Therefore, there is a big current necessity to developfaster, cheaper and more accessible sensing methods for the sensitive and selective detection of dangerous levels of metal ions. 

Lately, luminescent metal nanoclusters, especially gold nanoclusters (AuNCs), became considerably interesting among researchers due to their favorable features, such as intrinsic photoluminescence (PL), non-toxicity, large Stokes Shift, and low photo-bleaching [1,12,13]. The typical approach to synthesize AuNCs involves the reduction of Au^3+^ salts to Au^0^ in the presence of different molecular templates, for instance proteins [14,15,16], peptides [17,18], amino acids [19], DNA [20], purines [21], etc. Guided by the various templates, the Au atoms gradually aggregate and form AuNCs. What brings a huge advantage to AuNCs is their size and emission spectral position which can be varied depending on the capping ligand and reducing agent [4,22,23], making AuNCs widely applicable in different fields, such as molecular identification [24], biolabeling [25], catalysis [26], imaging [27], sensing [28], etc. Recently, great advances were made in the field of AuNCs-based sensing applications, as they present selective and sensitive quenching or enhancement of their PL when in contact with particular analytes [29,30]. For example, Li et al. reported an egg-white protein-coated AuNCs sensor for the selective detection of Fe^3+^, NO- and cystein [4]. Mu et al. prepared L-proline stabilized AuNCs for the colloidal detection of serum Fe ions [31] and Su et al. reported a sensor based on histidine (His) stabilized AuNCs for the detection of Fe ions in bean sprouts [32]. Even though there are a lot of progressions in the field of Fe monitoring and sensing, the reported detection methods rely on the use of heavy, sophisticated and expensive equipment as well as trained personal. For this reason, there is a great necessity to develop a cheap, fast, portable, highly sensitive and selective sensor for the on-site detection of pollutant Fe ions for an improved monitoring of the environmental conditions and human health in the future. 

Recently, paper is considered to be a high-profile candidate for the fabrication of novel lab-on-a-chip sensing platforms owing to its high efficiency-price ratio, portability, flexibility and eco-friendly properties. Specifically, the cellulose fiber component can be easily functionalized or impregnated with colloidal samples, thus resulting in attractive properties such as great hydrophilicity, permeability and reactivity [33,34]. Therefore, filter paper can be promising for various detection applications in environmental monitoring or food quality control [35].

Here, we successfully fabricated a novel efficient portable paper-based sensor for the selective and sensitive detection of Fe ions in real water samples based on the effect of PL quenching occurring for paper-incorporated histidine capped AuNCs (His-AuNCs). Firstly, we successfully synthesized photoluminescent His-AuNCs via a novel microwave-assisted approach. The transmission electron microscopy (TEM) images confirmed the small dimensions of the AuNCs, their average size being 2.9 ± 0.3 nm. The intrinsic PL signal from 471 nm exhibited by His-AuNCs under 380 nm excitation proved to be stable under constant irradiation and over time, while its intensity exhibited a slight dependence on temperature. Subsequently, the selectivity of His-AuNCs towards Fe^2+^ was proven by the PL quenching effect observed in colloidal solution. Regarding the sensitivity of the sensor, we found a great dependence between Fe^2+^ concentration and the AuNCs’ PL quenching, obtaining a linear dynamic range from 0.022 to 4.400 mM with a great correlation coefficient. The limit of detection (LOD) was obtained to be 0.2 μM, a much smaller value than the limit of Fe that is accepted by World Health Organization for drinking water (35 μM) [5]. Afterwards, the synthesized His-AuNCs were immobilized onto Whatman filter paper to achieve an easier-to-use, more efficient and accessible portable sensing device for detecting dangerous amounts of Fe ions, under UV light excitation, from low-volume water samples, based on the visual identification of PL quenching response. In order to quantify this sensing approach, photographic images were captured with a smartphone camera and the average blue intensity ratio (I/I_0_) of the His-AuNCs-paper spots was plotted against [Fe^2+^] which revealed a linear dynamic range between 9 and 97 µM with an exceptional correlation coefficient of 0.995. The LOD of the newly-proposed portable sensor is 3.2 µM. In addition, selectivity and competitivity assays demonstrate the high selectivity and accuracy of the proposed sensing platform towards the detection of Fe ions. Finally, we validated the as-prepared sensing platform for the detection of Fe ions from spiked (35 μM Fe^2+^) real water samples with recoveries ranging between 102.0% and 105.4%, proving the great accuracy of the sensor. To the best of knowledge, this represents the first demonstration of a AuNCs-paper-based sensing platform able to quantify dangerous levels of Fe^2+^ from water. Moreover, in view of future applications, digital images of the sensing platform can be taken by anyone using a smartphone camera and a pocket UV lamp and sent to environmental water-monitoring organizations. In conclusion, we successfully developed an efficient sensing platform based on His-AuNCs-impregnated paper that is fast, cheap, portable, accessible, qualitative and quantitative. The proposed ensemble represents a promising candidate as a lab-on-a-chip device in environmental applications for the sensitive and selective detection of dangerous levels of Fe ions from low-volume real water samples.

## 2. Results and Discussion

### 2.1. Characterization of His-AuNCs

**Figure 1:**
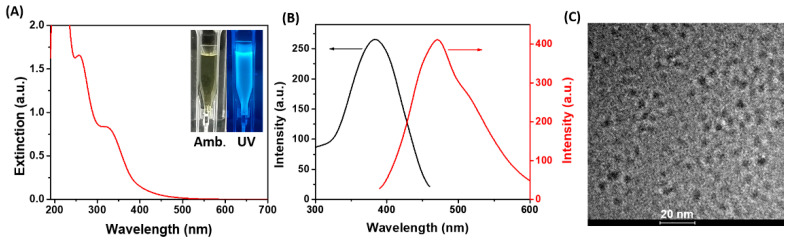
(**A**) The UV-Vis absorption spectrum of His-AuNCs (inset: images of His-AuNCs in colloidal suspension under ambient (Amb.) and UV light). (**B**) The excitation (black line, λ_em_ = 471 nm) and emission (red line, λ_ex_ = 380 nm) spectra of the synthesized His-AuNCs. (**C**) Representative HR-TEM picture of His-AuNCs.

After the microwave-assisted synthesis of His-AuNCs was completed, the colloidal solution presented a pale-green color in ambient light, while it turned to an intense blue color under UV light excitation (inset—Figure 1A), which represents a clear indicator that the as-synthesized His-AuNCs possess an intrinsic PL. 

The UV-Vis absorption spectrum (Figure 1A) presents two peaks at 256 and 324 nm which are typical for AuNCs stabilized by His according to the literature [36]. Moreover, the absence of the localized surface plasmon resonance band, typical for larger-sized Au nanoparticles, suggests that the as-prepared His-AuNCs are very small in size. Accordingly, the morphology of the newly prepared His-AuNC was investigated through HR-TEM. A representative image of our samples is presented in Figure 1C while the size histogram is shown in Figure S1. The average size was calculated to be 2.9 ± 0.3 nm, after analyzing more than 100 particles. Moreover, the His-AuNCs present a negative charged surface of −12 mV according to the zeta potential analysis (Figure S2). Further, the emissive properties of the His-AuNCs were evaluated. The colloidal His-AuNCs present an excitation maximum at 380 nm corelated with a strong PL emission at 471 nm. Presently, there is no concrete theory that explains why NCs exhibit intrinsic PL, yet the effect of metal confinement represents a generally recognized explanation throughout literature [37,38]. In summary, for metal nanostructures that have sizes close to the Fermi wavelengths of metals (2–3 nm), their continuous energy bands transform into discrete ones, enriching the nanostructures with molecular-like properties such as PL emission. Consequently, the emission of AuNCs is considered to originate from sp-d (interband) and sp-sp (intraband) transitions. Subsequently, compared to the emission of His-AuNCs synthesized following a literature procedure (Figure S3A—black line) [1], the optimized synthesis using microwaves showed a four-times stronger PL emission, under the same excitation (Figure S3A—red line). Additionally, under continuous 380 nm irradiation, the His-AuNCs exhibit 91.8% of their initial emission, implying a high photostability. Also, the PL of His-AuNCs remains stable even 3 months after their synthesis, as the spectra from Figure S3B prove. Next, the temperature-dependent behavior of the PL emission of His-AuNCs was investigated at various temperatures ranging between 25 °C and 50 °C, under 380 nm excitation. As illustrated in Figure S4, a slight decrease in PL intensity of 16% was observed at 50 °C compared to the one at 25 °C. However, the process is reversible in the particular temperature range (25–50 °C) as the PL returns to its initial intensity after the cooling process, demonstrating the high stability of His-AuNCs’ emission. Non-radiative deexcitation through collisions due to increased Brownian movement could be the possible explanation of the slight nonradiative relaxation of the His-AuNCs occurring with temperature increase. The aforementioned results demonstrated that the as-synthesized His-AuNCs present a strong and photostable PL, an essential property for sensing applications.

### 2.2. Performance of His-AuNCs-Based Colloidal Sensor

**Figure 2:**
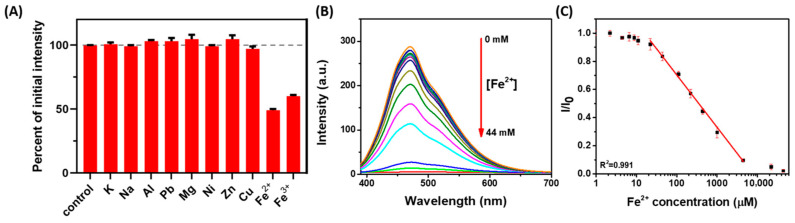
(**A**) The effect of quenching (I/I_0_) induced by the presence of common metal ions on the PL of His-AuNCs at 471 nm. (**B**) PL spectra of His-AuNCs in the presence of Fe^2+^ ions with concentration in the 0–44 mM range. (**C**) PL intensity ratio (I/I_0_) of His-AuNCs at 471 nm as function of [Fe^2+^] along with the linear fitting function used, revealing a linear dynamic range from 0.022 to 4.4 mM. λ_ex_ = 380 nm.

To uncover the selectivity of His-AuNCs towards Fe ions, His-AuNCs in colloidal solution were incubated with common metal ions (K^+^, Na^+^, Al^3+^, Pb^2+^ Mg^2+^, Ni^2+^, Zn^2+^, Cu^2+^, Fe^2+^, Fe^3+^) and their PL intensity at 471 nm was investigated under 380 nm excitation. The quenching effects induced by common metal ions such as K^+^, Na^+^, Al^3+^, Pb^2+^ Mg^2+^, Ni^2+^, Zn^2+^ and Cu^2+^ on the PL of His-AuNCs were found to be neglectable compared to the ones caused by Fe^3+^ and Fe^2+^, which had a strong quenching effect on their PL emission (Figure 2A). Specifically, the emission of His-AuNCs dropped to 49% of the initial emission in the presence of Fe^2+^ and to 60% in the presence of Fe^3+^, thus confirming that the proposed AuNCs-based sensor is highly selective towards Fe ions. Li et al. supposed that the mechanism for the observed PL quenching effect may be related with Fe inducing the aggregation of AuNCs at microscopic level [4]. The photoluminescence quenching effect is known to be due to the interaction between the AuNCs’ stabilizing agent and the detecting analyte [39]. This phenomenon could be a result of the coordination and cross-linking of the amino and carboxyl groups by Fe ions, since this phenomenon was previously observed [32,40]. In this work, the chelation between histidine and Fe ions results into an energy loss of the His-AuNCs’ excited electrons that is directly dependent on the number of ions that are bounded to the stabilizing agent and, therefore, inducing a PL quenching effect. 

In the following part of our study, we evaluated the sensitivity of the proposed sensor for the detection of Fe^2+^, in solution phase, based on the above observed strong PL quenching effect. The recorded PL spectra of His-AuNCs colloidal solutions incubated with different Fe^2+^ concentrations in the 0–44 mM range are shown in Figure 2B, while the corresponding plot of the PL quenching effect as a function of [Fe^2+^] is illustrated in Figure 2C. Following the corresponding fitting operations, the (I/I_0_) plot versus [Fe^2+^] reveals a wide linear dynamic range from 0.022 to 4.4 mM with an excellent correlation coefficient of 0.991. Furthermore, the limit of detection (LOD) was found to be 0.2 μM, which makes the colloidal sensor a relevant one for preventing the dangerous intake of Fe ions, as the limit of Fe in drinking water stands at 35 μM according to the World Health Organization [5]. Additionally, the relative standard deviation was calculated to be at a value as low as 2.1% for three assays attained for the detection of 22 μM Fe^2+^ levels, proving the high precision and reproducibility of the detection platform.

### 2.3. Fe^2+^ Sensing with His-AuNCs-Paper-Based Platform

**Figure 3:**
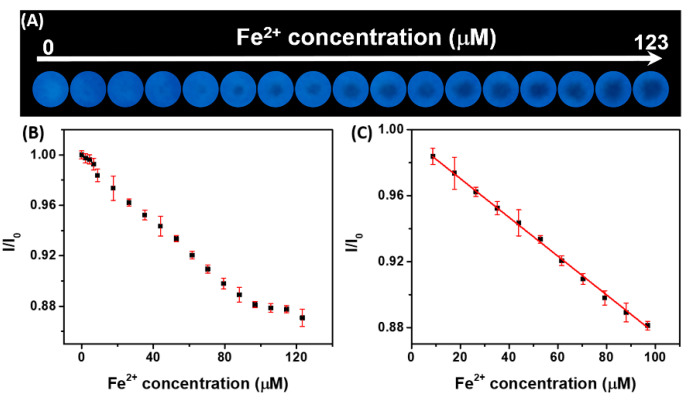
(**A**) Photographic pictures of His-AuNCs-paper-based sensor. The pictures were acquired under UV light excitation, 15 min after drops of solutions containing different concentrations of Fe^2+^ (0–123 μM) was added over the His-AuNCs-paper spots, previously dried for 24 h. (**B**) Plot of the average blue intensity value ratio (I/I_0_) of His-AuNCs-paper spot versus [Fe^2+^] in the 0–123 μM range. (**C**) Plot of the average blue intensity value ratio (I/I_0_) of His-AuNCs-paper spot versus [Fe^2+^] along with the linear fitting function used which reveals a linear dynamic range from 9 to 97 µM.

Even though His-AuNCs perform as an excellent sensing platform in solution, this detection method exhibits low portability, low accessibility, is time-consuming and requires the need of trained personal to perform the analysis. Therefore, the development of cheaper, easier, faster and portable sensing methods is highly desired. Lately, filter paper has been extremely exploited in the development of cheap sensing devices due to its excellent porosity, versatility, permeability and eco-friendly properties [33,34,35]. Therefore, we combined the appealing properties of filter paper with the excellent sensing capacity of His-AuNCs in order to fabricate a cheap and accessible portable device for the rapid, qualitative and quantitative detection of Fe^2+^ from water. Explicitly, the His-AuNCs- paper-based sensor was fabricated by drop-casting His-AuNCs onto Whatman filter paper and allowing it to dry as described in Section 3.5. Photographic pictures of different batches of the newly prepared sensing platform, taken under UV light after 1 day of storage are presented in the first row of Figure S5. After a thorough analysis of the images, no changes in intensity were observed between batches, demonstrating the high reproducibility of the developed sensing platform. Moreover, each batch exhibited the same intensity even after 7 days of storage, proving also the high photostability of the platform. Further, small drops of Fe^2+^ solutions of different concentrations in the 0–123 μM range were released over the His-AuNCs-impregnated paper sensing sites. The effect of PL quenching was investigated visually under UV light illumination after 15 min of drying at room temperature. Moreover, for a detailed examination of the PL quenching effect, a photographic image was acquired using a smartphone camera (Figure 3A).

In the reference His-AuNCs-paper spot, where we dropped only ultrapure water, no changes in color or PL intensity were observed (see first left spot from Figure 3A). Compared to the reference, the spots contaminated with various Fe^2+^ concentrations exhibit a detectable color change in the middle due to the effect of PL quenching, owing to the interaction between the His-AuNCs and the Fe^2+^ ions. Specifically, the PL quenching effect can be seen as a change in color from the intense blue in a gray-blackish spot in the middle of the detection area which grows in size as the tested [Fe^2+^] increases, which proves the PL turning-off effect and demonstrates thus the successful detection. Moreover, the plot of the average blue intensity ratio (I/I_0_) of the His-AuNCs-paper spots versus [Fe^2+^] in the 0–123 μM range is presented in Figure 3B. After the corresponding fitting assays, the obtained plot of the average blue intensity versus [Fe^2+^] revealed a linear dynamic range between 9 and 97 μM (Figure 3C) with an excellent correlation coefficient of 0.995, which represents, to the best of our knowledge, the first reports of a AuNCs-paper-based sensing platform able to quantitatively detect Fe^2+^. Furthermore, the LOD of the paper-based sensor was obtained to be 3.2 μM, proving that the sensor is successful in detecting relevant values of Fe^2+^, taking into consideration that levels of Fe over 35 μM are considered toxic [5]. The importance of this sensing approach comes from its accessibility, fast results and easiness, as anyone can perform the detection, take a photographic image of it and sent it to a water monitoring organization. Furthermore, we performed a selectivity assay on paper-based sensor. For this, common metallic ions at a concentration of 35 μM were dropped over the detection sites and photographic pictures of paper platform under UV illumination were recorded. Images are presented in Figure S6. After the interaction with the following metallic ions: K^+^, Na^+^, Al^3+^, Pb^2+^ Mg^2+^, Ni^2+^, Zn^2+^ and Cu^2+^, the sensing platform exhibits blue intensity levels similar to the control sample (water). However, the detection sites over which Fe ions were dropped (Fe^2+^ and Fe^3+^) exhibiting a photoluminescence quenching which can be easily observed by the naked-eye, demonstrating the high selectivity of the paper sensor towards both Fe ions. Moreover, we performed additional competitivity assay meant to reveal whether the proposed paper-based sensor is capable of selectively detecting the two types of Fe ions from water samples. Explicitly, we evaluated the sensor capacity to detect Fe^2+^ at a final concentration of 35 μM in the presence of different metallic ions (K^+^, Na^+^, Al^3+^, Pb^2+^ Mg^2+^, Ni^2+^, Zn^2+^, Cu^2+^, Fe^2+^, Fe^3+^) at the same final concentration in the analyzed sample, 35 μM. The obtained photographic images are presented in Figure S7. After analyzing the quenching effect of the detection sites (Table S1), recoveries between 90.5% and 104.0% were obtained in the presence of common metallic ions such as K^+^, Na^+^, Al^3+^, Pb^2+^ Mg^2+^, Ni^2+^, Zn^2+^ and Cu^2+^ proving once again the selectivity of the proposed sensor towards Fe, but also its high precision. Interestingly, the detection of Fe^2+^ in the presence of the same concentration of Fe^3+^ or Fe^2+^ returned recoveries of 195.1% and 200.2%, respectively, with a very similar quenching effect, which suggests the presence of a ~70 µM concentration in both cases. These results demonstrate that the proposed sensing platform is capable of selective and sensitive detection of the total amount of Fe ions present in the analyzed sample, however it cannot distinguish the type of Fe ion. Therefore, the portable and cheap paper-based sensing platform represents an efficient, sensitive and selective sensor that can be used in the future for the detection of Fe ions from low-volume water samples.

### 2.4. Fe Sensing from Real Water Samples with His-AuNCs-Paper-Based Platform

**Figure 4:**
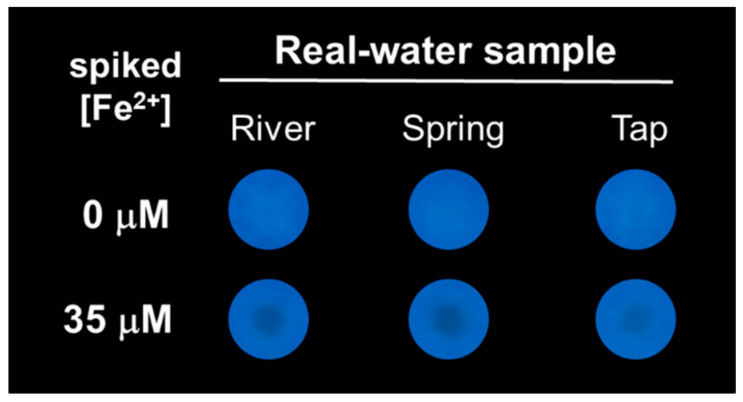
Photographic pictures of His-AuNCs-paper-based platform acquired during the Fe sensing assays (Fe^2+^ spikes) from selected local water samples. The pictures were acquired under UV light excitation, 15 min after the real water samples with and without the 35 μM Fe^2+^ spikes were dropped. The His-AuNCs-paper spots were previously dried for 24 h.

Since the paper-based sensor proved to have a sensitive response against Fe, we further evaluated the platform for the detection of Fe ions from local real water samples, in particular river, spring and tap water. The photographic images acquired under UV light corresponding to the His-AuNCs-paper spots, over which 5 μL of each real water samples were dropped, are presented in Figure 4.

For the tested real water samples, no traces of Fe were detected with our platform, thus, the sensing assays were further performed using the same samples spiked with 35 μM of Fe ions (i.e., Fe^2+^) (see spots in 2nd line from Figure 4). After a thorough naked-eye examination, a slightly gray-blackish spot was observed in the middle of the detection areas, similar to the one obtained by using a laboratory water sample with 35 μM of Fe^2+^. Moreover, the average blue intensity values of the spots were obtained via the ImageJ software and were used to calculate the actual concentration of Fe^2+^ detected by the proposed His-AuNCs-paper-based sensing platform, as presented in Table 1.

The obtained recoveries were between 102.0% and 105.4%, proving the good precision of the fabricated portable sensor. The aforementioned results demonstrate that the proposed paper-based platform is a promising candidate as an efficient lab-on-a-chip device with environmental applicability for the cheap and fast detection of dangerous levels of Fe ions from real water samples.

## 3. Materials and Methods

### 3.1. Chemicals

Histidine (His), Grade 4 Whatman^®^ qualitative filter paper (Whatman no. 4), hydrogen tetrachloroaurate (III) trihydrate (HAuCl_4_·3H_2_O), potassium chloride (KCl_2_), sodium chloride (NaCl), aluminum chloride (AlCl_3_), lead chloride (PbCl_2_), magnesium chloride (MgCl_2_), nickel chloride (NiCl_2_), zinc chloride (ZnCl_2_), copper chloride (CuCl_2_), iron (II) chloride (FeCl_2_) and iron (III) chloride (FeCl_3_) were acquired from Sigma-Aldrich. Local real water samples were collected from a local distributor (tap), spring and river sources. The reagents used in this work are all of analytical grade. A Milli-Q Millipore purification system from Merck (Darmstadt, Germany) was used to produce ultrapure water with a resistivity of 18 MΩcm used for the preparation of the solutions.

### 3.2. Synthesis of Colloidal His-AuNCs

All the glassware used in this work were carefully cleaned with aqua regia (HCl/HNO_3_, volume ratio 3:1) and rinsed thoroughly with ultrapure water before use. His-AuNCs were prepared using a novel method: His (0.1 M, 3 mL) was mixed with HAuCl_4_ (10 mM, 1.5 mL) into a G10 sealable microwave vessel and the mixture was introduced in a microwave reactor, where the probe was irradiated in pulses of 850 W power at 90 °C and 1200 rpm for 30 min. Afterwards, the His-AuNCs were purified by centrifugation at 6000 rpm for 10 min using a 10 kDa Satorious centrifugal concentrator from Hettich (Westphalia, Germany) to eliminate the surplus of His and unreduced ions. 

### 3.3. Selectivity Assay

The selectivity assays consisted in evaluating the PL intensity of His-AuNCs after interaction with common metal ions (K^+^, Na^+^, Al^3+^, Pb^2+^, Mg^2+^, Ni^2+^, Zn^2+^, Cu^2+^, Fe^2+^, Fe^3+^). Specifically, the colloidal solution of His-AuNCs (350 μL at initial synthesis concentration) was incubated with common metal chlorides solutions (350 μL at 1 mM concentration) for 15 min at room temperature. The PL quenching effect was analyzed using a JASCO spectrofluorometer under 380 nm excitation and the experiments were repeated three times.

### 3.4. Sensitivity Assay

The sensitivity of the His-AuNCs-based colloidal sensor towards Fe^2+^ was evaluated by incubating for 15 min the colloidal His-AuNCs (350 μL at initial synthesis concentration) with a solution containing Fe^2+^ at concentration ranging between 0 and 44 mM. Afterwards, the PL intensity of the His-AuNCs in the presence of different concentrations of Fe^2+^ was analyzed via a JASCO spectrofluorometer under 380 nm excitation. The degree of PL quenching was correlated with the Fe^2+^ concentration in order to obtain the calibration curve. The *LOD* was calculated using the following formula [41]:

(1) $$LOD=3.3\frac{S_{y}}{S}$$

where *Sy* is the standard deviation of the response and *S* represents the slope of the obtained calibration curve. The experiments were repeated three times.

### 3.5. Fabrication and Performance of His-AuNCs-Paper-Based Sensor

The His-AuNCs-paper-based sensor was obtained by drop-casting 5 μL of His-AuNCs onto Whatman filter paper, thus creating circular areas acting as active sensor sites. Afterwards, the His-AuNCs-paper-based sensor was dried at room temperature for 24 h in a dark place. Next, 5 μL of Fe^2+^ solution at concentrations in the 0–123 μM range were added onto the circular His-AuNCs-impregnated paper spots and left to incubate for 15 min. The PL quenching effect occurring in the circular areas was evaluated under UV light. Different-sized black spots occurred in the middle of the His-AuNCs-impregnated paper spots due to PL turning-off as function of Fe^2+^ concentration. Pictures were taken by a smartphone camera and investigated using ImageJ software by calculating the average blue value of the spot. The collected blue values were used to obtain a calibration curve for the detection of Fe^2+^ with the fabricated paper-based sensing platform. The LOD was calculated using Equation (1). The paper-based sensing platform’s selectivity assays consisted in evaluating the PL quenching effect of the incorporated His-AuNCs after interaction with different metallic ions (K^+^, Na^+^, Al^3+^, Pb^2+^ Mg^2+^, Ni^2+^, Zn^2+^, Cu^2+^, Fe^2+^, Fe^3+^) at a concentration of 35 μM. Moreover, a competitivity assay was performed by evaluating the quenching effect of samples containing 35 μM of Fe^2+^ in the presence of different metallic ions (K^+^, Na^+^, Al^3+^, Pb^2+^ Mg^2+^, Ni^2+^, Zn^2+^, Cu^2+^, Fe^2+^, Fe^3+^) at the same final concentration of 35 μM. The average blue intensity of the detection sites after the interaction with Fe^2+^ in the presence of different metallic ions was evaluated using ImageJ and divided to the average blue intensity of the control sample (water) to calculate the detected Fe^2+^ concentration using the calibration curve presented in Figure 3C. The experiments were conducted in triplicate.

### 3.6. His-AuNCs-Paper-Based Fe Sensing in Real Water Samples

To validate and demonstrate the relevance of the novel His-AuNCs-paper-based sensing platform, we analyzed different real water samples, from a local river, spring and tap waters. The tested water samples showed no traces of Fe ions; thus, they were spiked with the most relevant concentration of Fe^2+^ (35 μM) and the aforementioned calibration curve was used to test the detection capacity of the new paper-based sensing platform using the spike and recovery test. 

### 3.7. Instrumentation

His-AuNCs were prepared in a microwave reactor (Monowave 300, Anton Paar, Graz, Austria). The absorption spectra were obtained with a JASCO V-670 UV-Vis-NIR spectrophotometer (Tokyo, Japan) in a quartz cuvette of 2 mm from Helma and analyzed with the Spectra Manager program (JASCO). HR-TEM images were acquired with a Tecnai TEM microscope (Tecnai G2 F20 X-TWIN, FEI Company, Hillsboro, OR, USA) at 200 kV accelerating voltage. The zeta potential of His-AuNCs was measured using the Nano ZS-900 Zetasizer from Malvern. The fluorescence spectra were measured with a JASCO FP6500 spectrofluorometer (Tokyo, Japan) having 1 nm spectral resolution and using a 150 W Xenon lamp as excitation source. The fluorescence spectra of His-AuNCs were obtained with excitation and emission bandwidths fixed at 3 nm. Helma quartz cuvettes (5 × 5 mm) were used for all fluorescence measurements, while the obtained spectra were analyzed with the Spectra Manager software. All photos regarding the paper-based detection assays were captured in a dark room with a smartphone camera with the following settings: ISO at 100, speed at A 1/10, exposure value at −0.8 and white balance at A 4400 K.

## 4. Conclusions

In conclusion, we have successfully fabricated a novel, reliable, fast, cheap and accessible His-AuNCs-paper-based portable sensor for the selective and sensitive detection of dangerous levels of Fe from low-volume real water samples. First, we synthesized colloidal His-AuNCs using a novel microwave-assisted method, which exhibited an intense, photostable and slightly temperature-dependent PL emission at 471 nm under 380 nm excitation. Next, we proved the selectivity of the as-synthesized His-AuNCs towards Fe ions and we investigated the sensitivity of our sensor in solution, by evaluating the PL emission in the presence of different concentrations of Fe^2+^. The plot (I/I_0_) versus [Fe^2+^] revealed a linear dynamic range between 0.022 and 4.4 mM with an excellent correlation coefficient, while the LOD was 0.2 µM, a value lower than the maximum levels of Fe ions admitted by the World Health Organization in drinking water (35 µM). For increased applicability, we subsequently incorporated the His-AuNCs onto Whatman filter paper to obtain an easy-to-use and portable sensing platform for faster and easier detection of Fe ions from a low volume of samples, under exposure to UV light, by visually evaluating the PL quenching effect occurring for the paper-incorporated His-AuNCs in the presence of Fe^2+^. Furthermore, photographic images of the detection paper active sites were taken via a smartphone camera and were analyzed using the ImageJ software. By plotting the average blue intensity ratio (I/I_0_) of the His-AuNCs-paper spots versus [Fe^2+^], a linear dynamic range was revealed between 9 and 97 µM with a correlation coefficient of 0.995. Up to our knowledge, we report here for the first time a AuNCs-paper based sensing platform able to quantify dangerous levels of Fe^2+^ from low-volume samples. More importantly, we obtained a LOD of 3.2 µM. In addition, selectivity and competitivity assays demonstrate the high selectivity and accuracy of the proposed sensing platform towards the detection of Fe ions, regardless of their type, in the analyzed water sample. Finally, the fabricated portable sensor was validated for Fe sensing in real water samples (spring, river and tap) spiked with 35 µM Fe^2+^. The recoveries ranging between 102.0% and 105.4% demonstrate the good accuracy of our portable sensor. Therefore, we demonstrate that the novel His-AuNCs-paper-based portable sensing platform can be an excellent candidate as a lab-on-a-chip device for environmental on-site monitoring of dangerous levels of heavy-metals in real-water samples.

## Figures and Tables

**Table 1:** Recovery tests for Fe^2+^ spiked in real water samples.

Type of Water	Added (μM)	Found (μM)	Recovered (%)
River	0	ND ^1^	-
	35	36.9 ± 1.6	105.4 ± 4.5
Spring	0	ND	-
	35	36.2 ± 1.4	103.4 ± 4.0
Tap	0	ND	-
	35	35.7 ± 2.0	102.0 ± 5.7

^1^ ND: non-detectable.

## Data Availability

Not applicable.

## Supplementary Materials

The following supporting information can be downloaded at: https://www.mdpi.com/article/10.3390/ijms232012410/s1.
