# Supplementary material for: Photoluminescent Histidine-Stabilized Gold Nanoclusters as Efficient Sensors for Fast and Easy Visual Detection of Fe Ions in Water Using Paper-Based Portable Platform
Source: Int J Mol Sci. 2022 Oct 17;23(20):12410. doi: 10.3390/ijms232012410 (PMC9604042; doi:10.3390/ijms232012410)
Supplement: Supplementary file 1 [file ijms-23-12410-s001.zip › ijms-1951354-supplementary.pdf]

Electronic Supplementary Information

Photoluminescent histidine-stabilized gold nanoclusters as efficient sensors for fast and easy visual detection of Fe ions in water using paper-based portable platform

Alexandru-Milentie Hada[a,b,†], Markus Zetes[a,b,†], Monica Focsan[a], Simion Astilean[a,b], Ana-Maria Craciun[a,*]

[a]Nanobiophotonics and Laser Microspectroscopy Center, Interdisciplinary Research Institute in Bio-Nano-Sciences, Babes-Bolyai University, 42 T. Laurian Str., 400271, Cluj-Napoca, Romania

[b]Faculty of Physics, Babes-Bolyai University, 1 M. Kogalniceanu str., 400084, Cluj-Napoca, Romania

[†]These authors contributed equally

*Corresponding author: ana.gabudean@ubbcluj.ro

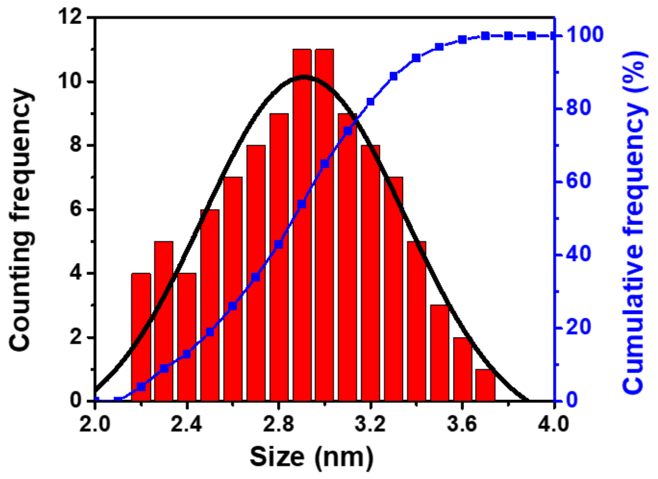

Figure S1. The size histogram of His-AuNCs with the corresponding counting and cumulative frequencies.

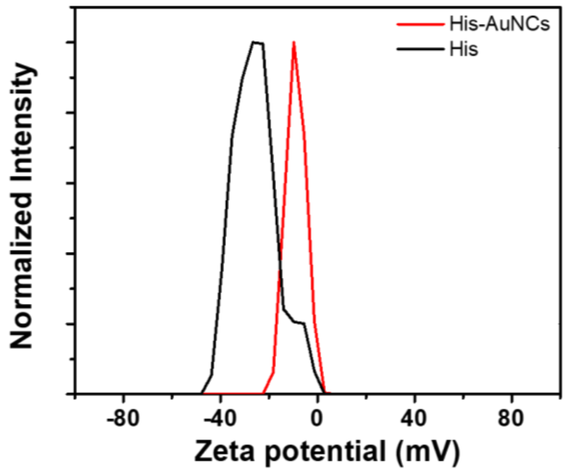

Figure S2. The zeta-potential of His and His-AuNCs.

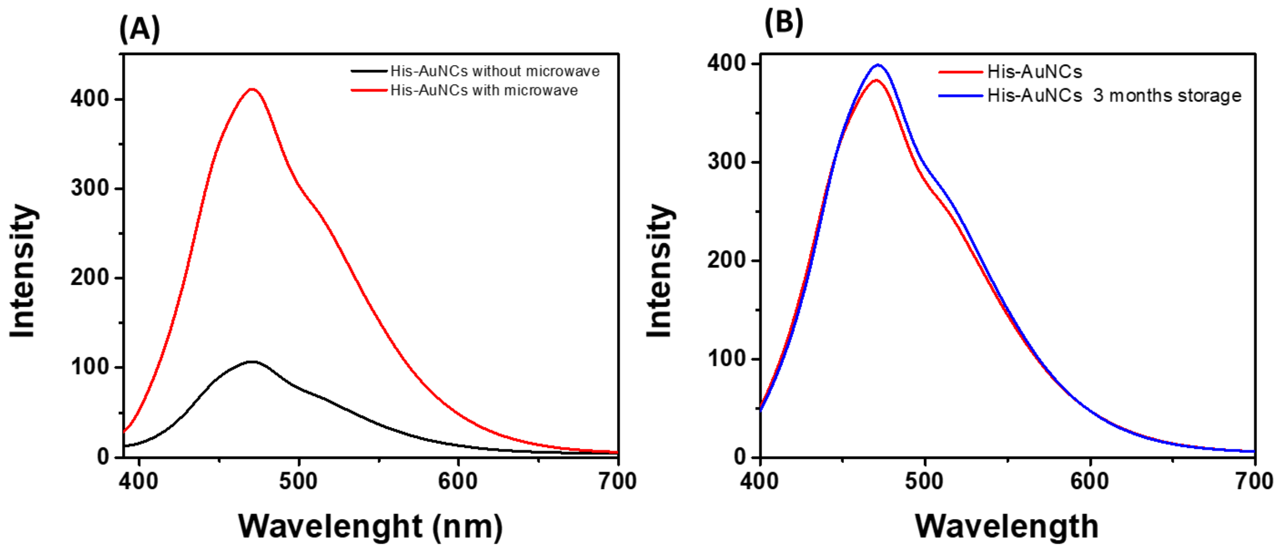

Figure S3. (A) The PL emission of His-AuNCs synthesized with and without microwaves. (B) The PL emission of His-AuNCs immediately after the synthesis and after 3 months of storage. Excitation wavelength: 380 nm.

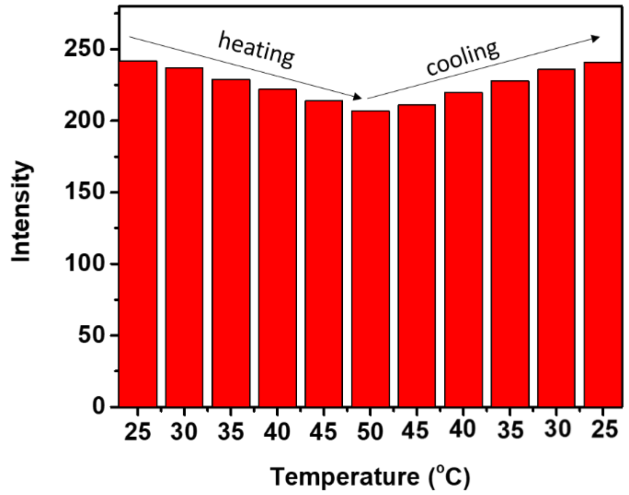

Figure S4. The temperature-dependent behavior of the His-AuNCs' PL. The intensity was recorded at 471 nm under 380 nm excitation.

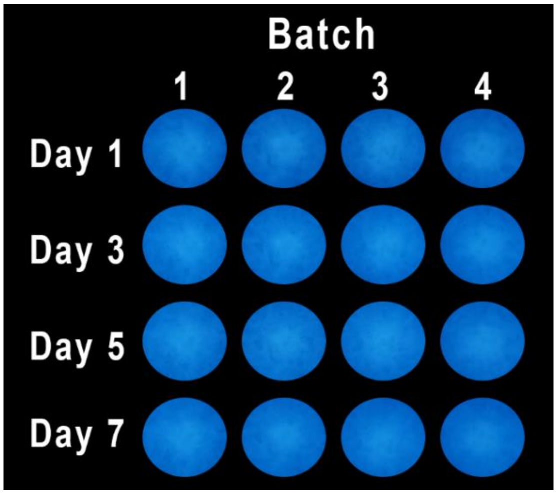

Figure S5. Photographic pictures of different batches of the paper sensing platform evaluated after 1, 3, 5 and 7 days of storage.

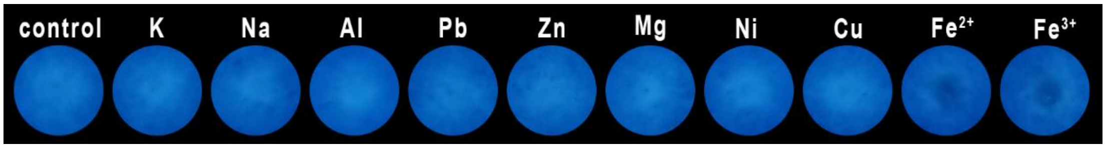

Figure S6. Photographic pictures of the paper sensing platform after interaction with common metallic ions (K, Na, Al, Pb, Mg, Ni, Zn, Cu, Fe^{2+}, Fe^{3+}) obtained in the selectivity assay.

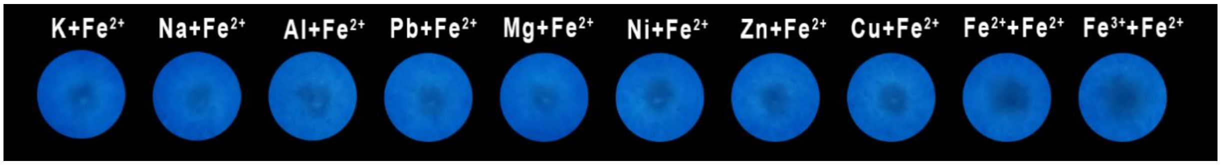

Figure S7. Photographic pictures of the paper sensing platform after interaction with a mixture of Fe^{2+} and different metallic ions (K, Na, Al, Pb, Mg, Ni, Zn, Cu, Fe^{2+}, Fe^{3+}) obtained in the competitivity assay.

Table S1. Recovery tests for Fe^{2+} (35 µM) in the presence of different metallic ions (35 µM) obtained after the analysis of the quenching effect observed in the photographic pictures from Figure S7.

Type of ion	Found (µM)	Recovered (%)
K	34.1 ± 0.7	97.4 ± 2.0
Na	33.5 ± 0.4	95.7 ± 1.1
Al	36.4 ± 0.7	104.0 ± 2.0
Pb	34.5 ± 0.5	98.5 ± 1.4
Mg	31.7 ± 0.7	90.5 ± 2.0
Ni	34.7 ± 0.7	99.1 ± 2.0
Zn	34.3 ± 0.8	98.0 ± 2.2
Cu	33.1 ± 1.01	94.5 ± 2.9
Fe^{2+}	70.1 ± 1.3	200.2 ± 3.7
Fe^{3+}	68.3 ± 1.1	195.1 ± 3.1
